# Correction: Context-dependent modification of PFKFB3 in hematopoietic stem cells promotes anaerobic glycolysis and ensures stress hematopoiesis

**DOI:** 10.7554/eLife.104576

**Published:** 2024-10-22

**Authors:** Shintaro Watanuki, Hiroshi Kobayashi, Yuki Sugiura, Masamichi Yamamoto, Daiki Karigane, Kohei Shiroshita, Yuriko Sorimachi, Shinya Fujita, Takayuki Morikawa, Shuhei Koide, Motohiko Oshima, Akira Nishiyama, Koichi Murakami, Miho Haraguchi, Shinpei Tamaki, Takehiro Yamamoto, Tomohiro Yabushita, Yosuke Tanaka, Go Nagamatsu, Hiroaki Honda, Shinichiro Okamoto, Nobuhito Goda, Tomohiko Tamura, Ayako Nakamura-Ishizu, Makoto Suematsu, Atsushi Iwama, Toshio Suda, Keiyo Takubo

**Keywords:** Mouse

Watanuki S, Kobayashi H, Sugiura Y, Yamamoto M, Karigane D, Shiroshita K, Sorimachi Y, Fujita S, Morikawa T, Koide S, Oshima M, Nishiyama A, Murakami K, Haraguchi M, Tamaki S, Yamamoto T, Yabushita T, Tanaka Y, Nagamatsu G, Honda H, Okamoto S, Goda N, Tamura T, Nakamura-Ishizu A, Suematsu M, Iwama A, Suda T, Takubo K. 2024. Context-dependent modification of PFKFB3 in hematopoietic stem cells promotes anaerobic glycolysis and ensures stress hematopoiesis. *eLife* **12**:RP87674. doi: 10.7554/eLife.87674.

Published 4 April 2024

We received a comment from a reader requesting clarification regarding which of the two G0 marker mouse models from the Kitamura Laboratory references was used in our study. To clarify this point, we have revised the ‘Mice and genotyping’ section of the Materials and Methods as follows: The original sentence,

‘mVenus-p27K- mice (17–20 weeks old) were provided by Kitamura Laboratory and used for cell cycle analysis (Fukushima et al., 2019; Oki et al., 2014). Mice were genotyped using PCR-based assays of tail DNA or transdermal EGFP fluorescence,’ has been updated to:

‘mVenus-p27K- mice (17–20 weeks old) were provided by Kitamura Laboratory and used for cell cycle analysis (Fukushima et al., 2019). Mice were genotyped using PCR-based assays of tail DNA or transdermal Venus fluorescence.’

Additionally, Oki et al.’s reference has been removed, as it is no longer cited in the revised text.

The article has been corrected accordingly.

